# Interventions to Drive Uptake of Voluntary Medical Male Circumcision—A Collection of Impact Evaluation Evidence

**DOI:** 10.1097/QAI.0000000000001155

**Published:** 2016-10-06

**Authors:** Sema K. Sgaier, Jason B. Reed, Maaya Sundaram, Annette Brown, Eric Djimeu, Renee Ridzon

**Affiliations:** * Surgo Foundation, Washington, DC;; † Department of Global Health and Population, Harvard T. H. Chan School of Public Health, Boston, MA;; ‡ Department of Global Health, University of Washington, Seattle, WA;; § Jhpiego, Washington, DC;; ‖ Bill & Melinda Gates Foundation, Seattle, WA;; ¶ FHI 360, Washington, DC;; # International Initiative for Impact Evaluation (3ie), Washington, DC; and; ** Boston University School of Public Health, Boston, MA.

**Keywords:** HIV, Africa, voluntary medical male circumcision, demand generation, impact evaluation, financial compensation, VMMC

In the last few decades, significant progress has been made in improving population health outcomes. This is largely attributable to the development of effective public-health tools—diagnostics, vaccines, other prevention technologies, and treatment regimens. Advances in delivery have enabled these technologies and interventions to reach end users in even the most remote areas. However, although uptake of services has followed, the pace has often been suboptimal. The global health field has come a long way in ensuring that services are available, but when it comes to motivating people to be better consumers of treatment and of preventive health services, there are relatively few proven interventions that promote uptake.

Voluntary medical male circumcision (VMMC) is one program facing such a challenge in eliciting uptake of services among large numbers of men and boys.^1^ VMMC is being scaled up in 14 eastern and southern African countries as a key HIV prevention intervention.^2^ To date, more than 11.7 million circumcisions have been performed, against a target of 20 million by 2016.^3^ A large proportion who have received services are adolescents,^4^ but for the VMMC program to achieve its intended rapid impact on the HIV epidemic, a greater proportion of older, sexually active, and high-risk males must access services.

The public health field needs new approaches and interventions to attract and motivate men to be circumcised. A systematic approach and framework to improve demand for VMMC has been recommended.^5^ The 4 components of this approach are (1) a greater emphasis on generating insights into the factors that drive or limit men's motivation and ability to undergo VMMC, (2) developing an innovative and comprehensive portfolio of demand generation solutions based on these insights, (3) implementing a proven portfolio of interventions at scale and in a coordinated manner to achieve high levels of coverage, and (4) a robust measurement and evaluation agenda. A key component of this approach is thus to generate evidence on which interventions work and should be scaled up to drive greater efficiency and effectiveness within programs. Equally important is knowing which behavior change interventions are not effective. Only a few evaluations of VMMC demand generation interventions have been published to date, which highlights the need for greater evidence.

The collection of impact evaluations in this supplement presents a rich and unique body of evidence on the effectiveness of various demand generation interventions for VMMC. It also provides an opportunity to analyze the science of evaluation in relation to demand generation interventions within the context of large-scale national programs. The approach taken to design and implement these interventions is interesting and worth highlighting. The International Initiative for Impact Evaluation (3ie), a grant-making nonprofit organization that promotes evidence-based policy making using impact evaluation, played a funding, convening, and technical support role. 3ie convened a group of evaluators, country stakeholders, and program implementers in a “matchmaking” workshop in April 2013.^6^ Teams were formed to work on intervention design, such that the interventions were field-driven and realistic, yet also included an evaluation component. The objective was to ensure rigorous assessment of impact and that effective interventions could be translated into large-scale programs and integrated into national VMMC policies and practices. The subsequent request for proposals produced 7 pilot interventions to increase the demand for VMMC with embedded qualitative and impact evaluations to determine whether and to what degree these interventions were effective. The program was designed to fund low-cost, rapid impact evaluations of the pilot interventions. Here, we synthesize and reflect on the approaches taken and the findings, in their relevance both to VMMC and to a broader array of large-scale global health programs.

## HOW TO, OR NOT, DRIVE VMMC UPTAKE?—INSIGHT FROM THE STUDIES

This collection includes 7 impact evaluations conducted in 6 of the 14 VMMC priority countries, one qualitative study and two analyses of methods. Table 1 lists the evaluation studies and includes 2 additional relevant studies not included in this supplement, which complement or help us interpret the findings from studies. The VMMC demand generation interventions addressed different possible barriers and potential facilitators influencing men's decisions to undergo VMMC as identified by acceptability studies.^7^ The possible barriers include fear of pain or adverse events, a period of sexual abstinence and threats to masculinity, opportunity cost of procedure in the form of lost wages from undergoing the procedure, and religious and cultural norms. Potential facilitators include incentives, delivery of information, social pressure, and peer and intimate partner influence. Each pilot intervention was designed to address one or more of these barriers and facilitators. Interventions explored both behavior change communication and compensation, including conditional compensation (direct economic compensation and lottery-based rewards), providing information using influencers (use of intimate partners to inform and encourage men, peers, and male role models in sports-based interventions), messages delivered through cell phones, and information postcards that explored message framing. Most of the interventions targeted males 18 years and above because reaching males of this age has been a challenge in most countries.

Two interventions (in Kenya and South Africa) offered direct fixed financial compensation to men seeking circumcision at clinics.^8,9^ In the South Africa intervention, to avoid risk of coercion, compensation was based on VMMC counseling only and not MC, although over 90% underwent the procedure. Both studies found a positive and statistically significant effect on uptake of circumcision from fixed financial compensation. An earlier study also in Kenya also found a positive effect of food vouchers on VMMC uptake.^10^

An in-depth qualitative study that explored how incentives influenced decision making in the Kenya intervention is especially insightful given concerns about the potential coercive effect of economic incentives.^11^ The study found that compensation addressed the major structural barrier of loss of wages and motivated men to act on the decision they had already made to undergo circumcision. The compensation did not result in uptake by men who were not interested in circumcision, showing that compensation did not adversely affect the voluntary nature of the procedure. Men reported several reasons for not responding to incentives—the amount was perceived as too low or insufficient to offset opportunity costs, or there were other barriers not addressed by the monetary compensation. These studies provide additional evidence that fixed compensation can increase uptake of VMMC without influencing decisions in a coercive way.

In contrast to the fixed compensation interventions, the 2 interventions that provided material incentives through a lottery (Kenya and Tanzania) did not produce statistically significant effects.^9,12^ The hypothesis behind these interventions was that lotteries introduce an element of risk that might appeal to people who engage in higher risk behavior, such as men who engage in high-risk sex. It relies on the principle that people generally overestimate their chance of winning big prizes.

One intervention in Zambia that built in peer influence provided a small financial reward to men who referred other men for circumcision; however, the intervention did not produce a significant effect in this study.^13^

Building on the theory of social learning and using peer influence, sports-based interventions provide a possible vehicle for behavior change. A previous evaluation of a 60-minute, interactive, soccer-themed educational intervention, bundled with cell phone message reminders, referral cards, and educational posters, with males 18 years and older in Zimbabwe [Male Circumcision Uptake through Soccer (MCUTS)] showed a weak association.^14^ The intervention reported in this supplement was the same soccer-based intervention used in MCUTS, but with additional logistical and behavioral follow-up, and targeted this time at adolescent males.^15^ This study found a larger effect than for MCUTS and included a qualitative component to tease out which parts of the intervention package led to the observed change in VMMC uptake. In the evaluation of this study, the approach was found to be highly acceptable with participation and personal interaction of the soccer coach as a key factor associated with uptake. Participants valued the coaches' discussion of their personal experiences with VMMC and willingness to accompany the boys to the VMMC clinic.^16^

Educational messaging to individual clients was examined in 2 studies. The SMS intervention in Zambia, which provided both conventional and client-tailored cell phone messages, did not show any effectiveness.^17^ The information-only postcards in South Africa also found no impact on VMMC uptake.^8^ These findings are likely due to the fact that lack of information about VMMC and its protective effects is no longer a major barrier to uptake and the information provided did not add to existing client knowledge. In fact, knowledge about VMMC is relatively high in most of the 14 VMMC priority countries because of many years of information campaigns. However, the challenge message (“Are you tough enough?”) did produce a positive and statistically significant effect, although much smaller than the effect from compensation.

It has been hypothesized that female partners of men could play a role in their decision making to get circumcised. A natural experiment intervention in Uganda was implemented whereby pregnant women in their third trimester were recruited at antenatal clinics and empowered with a package of comprehensive information about VMMC and trained in negotiation skills.^18^ The timing was chosen because of the unique opportunity offered by childbirth to align the periods of sexual abstinence postpartum and postcircumcision. However, the intervention did not prove to be effective for increasing uptake. While most women in the intervention group did deliver the VMMC message, many shared mixed experiences, and two-thirds did not feel comfortable engaging their partners in the conversation. A separate intervention in South Africa using “information postcards” to some degree also explored the role of women. The postcards distributed to men stated that a recent survey indicated that among partners of uncircumcised men, 2 of 3 would prefer that their partners be circumcised, in addition to providing basic information on the protective effect of VMMC. The effect size was small and statistically insignificant for the intervention. These studies highlight the need for greater evidence on the potential role of women in men's decision-making, and in combination with findings from the other interventions perhaps indicate that male peers might be more effective channels of influence.

## REFLECTIONS AND LESSONS FROM THE COLLECTION

This collection of impact evaluations and associated analysis provides a rich and unique body of evidence on the effectiveness of various demand generation interventions for VMMC. The study findings suggest that financial compensation designed to relieve the opportunity or transportation costs associated with undergoing the procedure can increase the uptake of VMMC. There is also evidence that programs using peer influence can be effective, although so far only sports-based programs demonstrate a strong effect.

Even among the effective demand generation interventions presented, the largest effect sizes were 7.6 and 7.1 percentage-point differences in uptake (for interventions in Zimbabwe and Kenya, respectively). These differences were statistically significant, but uptake rates are low considering the goal of 80% circumcision coverage.

Whether interventions such as these can make a big difference in coverage may depend on several factors. First, the interventions were evaluated for their impact after only a short period (in most cases 3 months, Table 1). However, a recent unpublished study suggests that for some men, it may take up to 2 years from the time of learning about VMMC to circumcision, so the short intervention period in these studies may be insufficient to result in uptake for many men. Second, it is possible that the barrier addressed by the intervention is not a significant one—for example, information on the benefits of VMMC may not address a barrier, since by now, most men are aware of these. Third, the different interventions evaluated here address barriers at different stages during the decision-making process for circumcision. From this standpoint, each intervention targets just the subset of men at the applicable stage for the intervention. Likewise, the above-mentioned factors may have also contributed to the low levels of uptake of the interventions that were not found to be effective. As different men have different barriers, there is a need for a combination of demand-side interventions to drive high levels of uptake of VMMC.

**TABLE 1.**
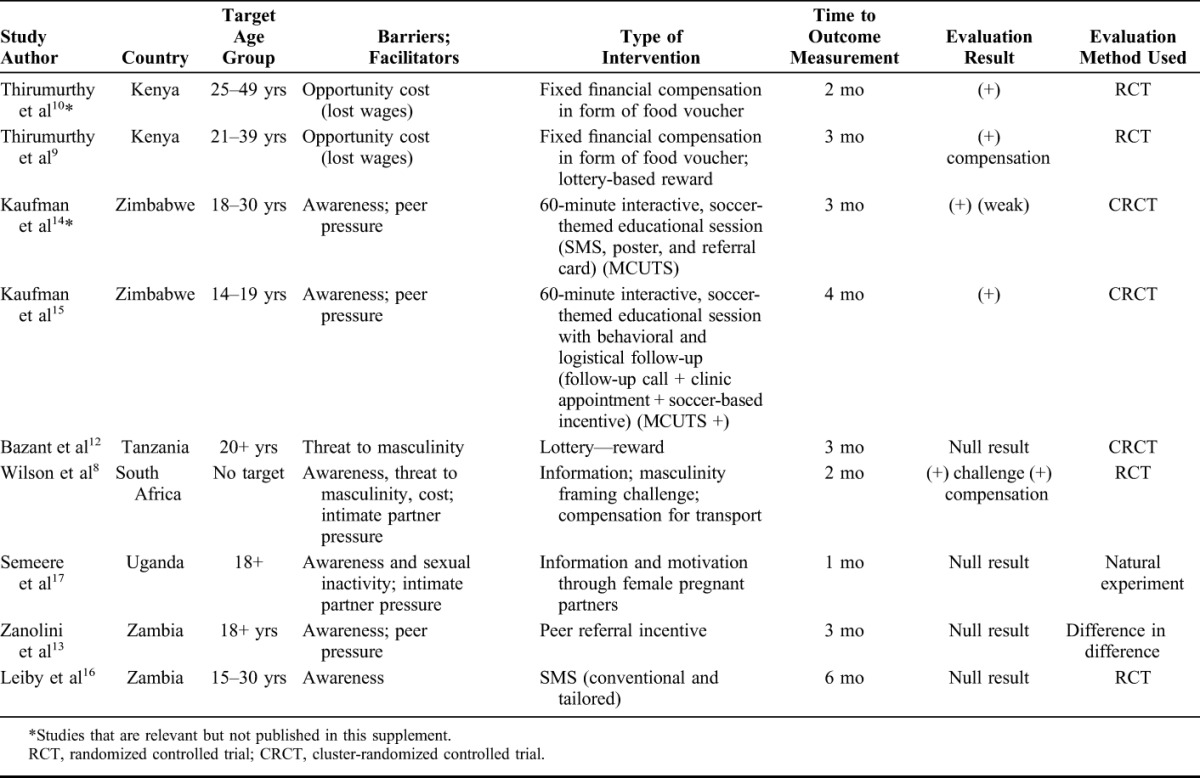
Summary of VMMC Demand Generation Impact Evaluations

Overall, these studies also highlight the need for more comprehensive, innovative, and context-specific insight generation work.^5^ The design of many of these interventions would have benefitted from a much richer and context-specific body of evidence, which currently does not exist.

**Lessons Learned From VMMC Demand Generation Interventions**

- Stakeholder engagement is crucial from design to implementation to ensure acceptability, context-driven design, and successful translation and integration of results into programs.
- Intervention design should be based on context-specific barriers and enablers that influence how the local population makes choices about health services.
- Effect sizes of the individual intervention evaluations can be generally small, highlighting the need for (1) longer intervention evaluation times and (2) evaluating a portfolio of interventions that address a comprehensive set of barriers affecting the decision-making of the target population.
- Qualitative components integrated into impact evaluations can provide important data that uncover “why” interventions do or do not work.
- Offering financial compensation appeared not to have a coercive effect on men's willingness to undergo VMMC.
- In addition to randomized controlled trials, there are other evaluation methods that can be used to evaluate interventions to drive demand that take into account the complex and evolving nature of programs.

The studies presented here underscore the value of coupling impact evaluations with qualitative components to uncover the underlying mechanisms that help explain the observed results. For example, the qualitative data that accompanied the Kenya fixed-financial compensation intervention highlight the noncoercive nature of incentives and show which groups of men responded to this intervention. In Uganda, the qualitative data indicated that empowering women with messages was not enough because many did not feel comfortable in engaging in conversation with their male partners.

The evidence provided here is a first step. Interventions that show significant impact on circumcision uptake will need to be introduced at scale and further evidence collected on how they affect circumcision uptake levels in combination with all other program elements, old and new.

Programs are complex, fluid, dynamic, and ever-changing. The contextual and programmatic changes may diminish or enhance the effects of interventions seen in the experimental evaluation studies. This calls for a different measurement approach—one that is more integrated within program and is ongoing.

How relevant are these country-specific results for other VMMC programs in the 14 prioritized countries? Context, intervention implementation, and target population, among many other factors, collectively affect the results yielded by a specific intervention within a larger public-health program. Programs should evaluate the potential impact of these interventions in their particular context. Given previous impact evaluative evidence, we suggest a rapid and implementation science approach to testing.

This collection represents one of the most comprehensive sets of studies addressing demand-side challenges in a large-scale public-health program. Although the interventions tested are specific to VMMC, the approaches used and lessons learned could be applied to other public-health programs. Along with evidence from other fields, these evaluations contribute to the body of evidence on different types of interventions, such as use of financial compensation, helping the community develop a more nuanced understanding of the circumstances in which they become a useful tool to drive uptake of services. Further innovation for demand-side interventions could benefit from comprehensive and context-specific research to understand the behavioral and psychological barriers and triggers for VMMC, and to uncover the motivations, aspirations, and decision-making processes of men regarding circumcision. These findings could inform the design of not a single, but rather a comprehensive portfolio of demand generation interventions that would collectively encourage a much larger group of men toward circumcision.
